# Changes in the soundscape of Girona during the COVID lockdowna)

**DOI:** 10.1121/10.0004986

**Published:** 2021-05-21

**Authors:** Rosa Ma Alsina-Pagès, Pau Bergadà, Carme Martínez-Suquía

**Affiliations:** 1 Grup de Recerca en Tecnologies Mèdia, La Salle-URL, C/Quatre Camins, 30, 08022 Barcelona, Spain; 2 Wavecontrol, c/Pallars, 65-71, 08018 Barcelona, Spain

## Abstract

The lockdown measures in Spain due to COVID-19 social measures showed a wide decrease in the urban noise levels observed. This paper presents an analysis of the noise levels in Girona, a 100 000 citizen city in the North-East of Catalonia (Spain). We present the *L_Aeq_* levels in four different locations from January 2020 to June 2020, including all the stages of the lockdown. Several comparisons are conducted with the monitoring data available from the previous years (2019, 2018, and 2017, when available). This analysis is part of the project “Sons al Balcó,” which aims to draw the soundscape of Catalonia during the lockdown. The results of the analysis in Girona show drastic *L_Aeq_* changes especially in nightlife areas of the city, moderate *L_Aeq_* changes in commercial and restaurants areas, and low *L_Aeq_* changes in dense traffic areas.

## I. INTRODUCTION

Environmental noise has been proved to cause more than 12 000 deaths in Europe per year, as the World Health Organisation (WHO) states (WHO, 2020). It generates more than 48 000 new cases of ischemic heart diseases, chronic annoyance to more than 22 × 10^6^ people (Guski *et al.*, 2017), and even chronic sleep problems to more than 6.5 × 10^6^ people (Blanes *et al.*, 2017). WHO declared the COVID-19 pandemic as an emergency on the 30th of January of 2020 (Jee, 2020), and several European countries developed lockdown plans, based on restricting commercial activities and flights, decreasing ground transportation (Aletta and Osborn, 2020), the closure of schools, and promoting teleworking, in order to minimize people contact, and hence, less contagion. Spain also had its own lockdown plan, starting in March and finishing in June, with several different stages (Alsina-Pages and Bergadà, 2021).

This social, educational, and industrial lockdown had a relevant impact on the cities' soundscape (Aletta *et al.*, 2020b; Basu *et al.*, 2020; Vida Manzano *et al.*, 2021). Most of the noise (Asensio *et al.*, 2020a) associated with everyday outdoor activities was severely decreased. Noisy ground transportation, mainly traffic noise (Aletta *et al.*, 2020a; Alsina-Pagès *et al.*, 2020a; Benocci *et al.*, 2020; Munoz *et al.*, 2020; Said *et al.*, 2020), railway noise, but also port noise (Čurovič *et al.*, 2021), airport noise (Vogiatzis *et al.*, 2020; Montano and Gushiken, 2020), industry noise (Mandal and Pal, 2020), and leisure-related noise (De Lauro *et al.*, 2020), were clearly reduced in the analyzed cities (Asensio *et al.*, 2020b; Bartalucci *et al.*, 2020), and even in quiet residential areas (Sakagami, 2020).

There have been several initiatives to track the soundscape changes by means of perception, questionnaires to citizens, and citizen science recordings, as in the United Kingdom—the Quiet Project (KSG Acoustics, 2020, Dance and Mcintyre, 2020), France (Bruit, Paris, France, 2020), Italy (AIA, 2020; Locate Your Sound, 2020; Scienzia sul Balcone, 2019)—even by means of questionnaires—(Bartalucci *et al.*, 2021), Barcelona Ajuntament de Barcelona, New York City NY Times Covid 19 (Bui and Badger, 2020), Turkey (Şentop Dümen and Şaher, 2020), and even aiming worldwide (Cities and Memory, 2020). In Catalonia, our project “Sons al Balcó” (Alsina-Pagès *et al.*, 2020b, 2021) is studying the effect of the lockdown on the perception of street noise, asking citizens to fill in a perception questionnaire and upload a short video recorded from their own home. In this work, we only focus on quantitative data, which comes from four out of the eight calibrated sensors deployed in the streets of Girona (Spain). We deeply analyze the data gathered in four sensors during the first six months of 2020 and compute the mean values gathered in 2019, 2018, and 2017 when available, in order to compare the street *L_Aeq_* levels among the four years and to evaluate the progression of the soundscape during the different stages of the confinement. Several conclusions about the different noise sources (traffic, leisure noise, tourism, etc.) are reached from this deep analysis of the sensors outcomes.

## II. SENSORS LOCATION AND DATA COLLECTION

### A. Locations in Girona and deployed devices

**FIG. 1.**
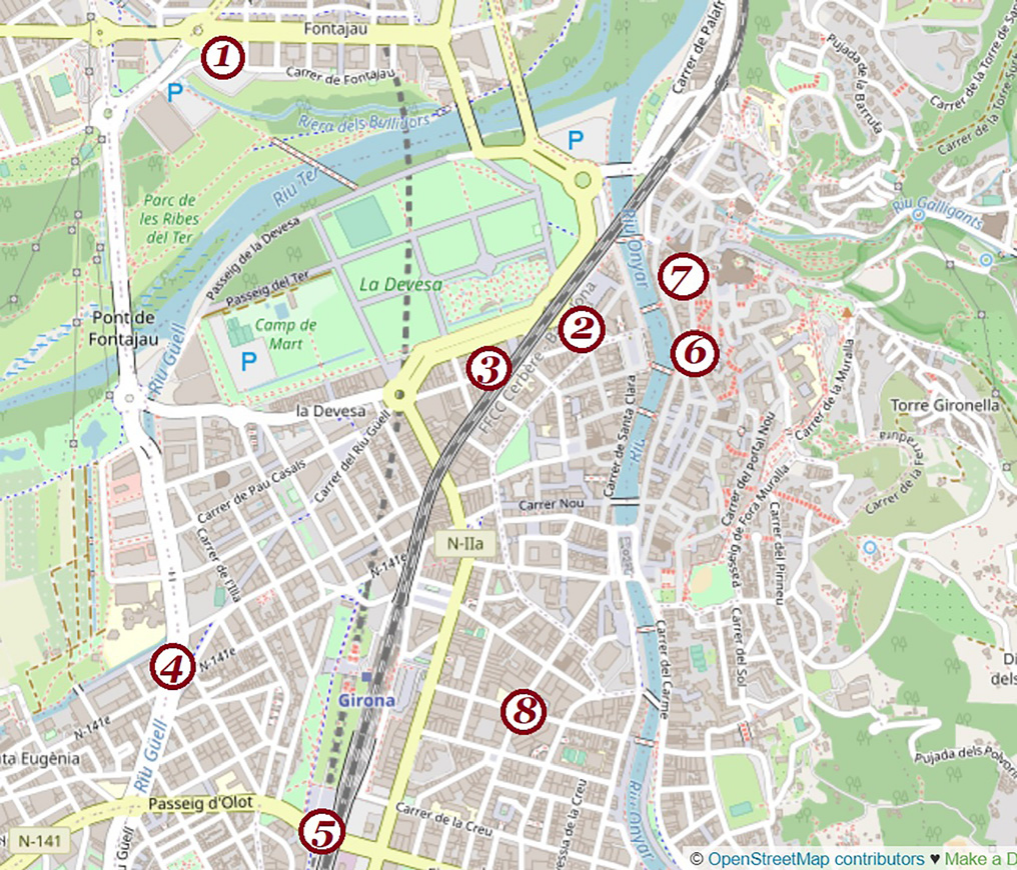
(Color online) Location of the eight sensors in the city center of Girona (OpenStreetMap, 03/01/2020).

In 2014, Girona City Council began the execution of an environmental plan with the installation of sound level meters, included in the project *Smart Green*. One of the actions was the deployment of a Noise Surveillance Network in the streets, including the installation of several sound sensors. The sensors deployed in Girona are located in the city center,^1^ and constitutes a network of up to eight sensors, which is detailed in Fig. 1. The *Visor Acústic* allows all citizens to check for the noise levels updated every hour in all the different parts of the city. The sensors locations are: (1) Rambla Xavier Cugat, (2) Avinguda Ramon Folch, (3) Carrer Figuerola, (4) Carrer Güell, (5) Passeig d'Olot, (6) Pujada de Sant Feliu, (7) Plaça de Sant Feliu, and (8) Carrer Joan Maragall w. Bisbe Lorenzana.

The device chosen can be installed in the streetlights and measures, with quality, the sounds over 40 dBA and up to 90 dBA, with an accuracy of ±2 dBA. They are precise enough to detect situations of high noise, which can cause annoyance to neighbors. The sensors have been deployed by Urbiotica^2^ and the signal processing corresponding to the equivalent levels evaluation has been coded by Keacoustics.^3^ The sensors give a detail of *L_Aeq_* with a maximum temporal resolution of 1 min. The sensors collect data all day and night and, besides several technical issues that occurred particularly during the lockdown, the analyzed data is continuous 24 h per day, throughout all analyzed weeks.

### B. Stages of the 2020 Lockdown in Spain

The lockdown in Girona was divided into five stages, as stated in (Asensio *et al.*, 2020b); the different stages require degrees of restrictions:

• Stage 1: 12/03/2020–13/03/2020—School suspended and telework suggested.
• Stage 2: 14/03/2020–28/03/2020—School, non-essential shops, and any events closed, no walking outdoors, telework unless justified.
• Stage 3: 29/03/2020–12/04/2020—School, non-essential shops, and any events closed, no walking outdoors, telework unless justified. Non-essential movement banned.
• Stage 4 (similar to Stage 2): 13/04/2020–26/04/2020—School, non-essential shops, and any events closed, no walking outdoors, telework unless justified.
• Stage 5: 27/04/2020–24/05/2020—School and any events closed, telework unless justified. Walks allowed (major restrictions).
• Stage 6: 25/05/2020–07/06/2020—School and any events closed, telework unless justified. Walks allowed (minor restrictions).

The weekly updates of the stages are used to label the figures of the analysis of the *L_Aeq_* in the four sensors. Just for clarification, we have to assume that there are slight variations of those stages, especially in small parts of the country where the evolution of the pandemic was not the expected.

## III. RESULTS OF THE EVALUATION

After a preliminary approach (Alsina-Pages and Bergadà, 2021), and considering the data issues that impact the completeness of the values of the noise levels, four sensors out of eight were chosen to conduct this analysis. Points #2, #4, #6, and #7 were chosen for several reasons: (i) the completeness of the data during the lockdown and the week previous to it, (ii) the completeness of the data the previous years during the same months of the lockdown, and (iii) the implication of several urban activities in the sound gathered in the selected sensors—traffic, railway, commerce, restaurants, leisure, etc. After a first analysis, four of the eight sensors accomplish these three requirements.

**FIG. 2.**
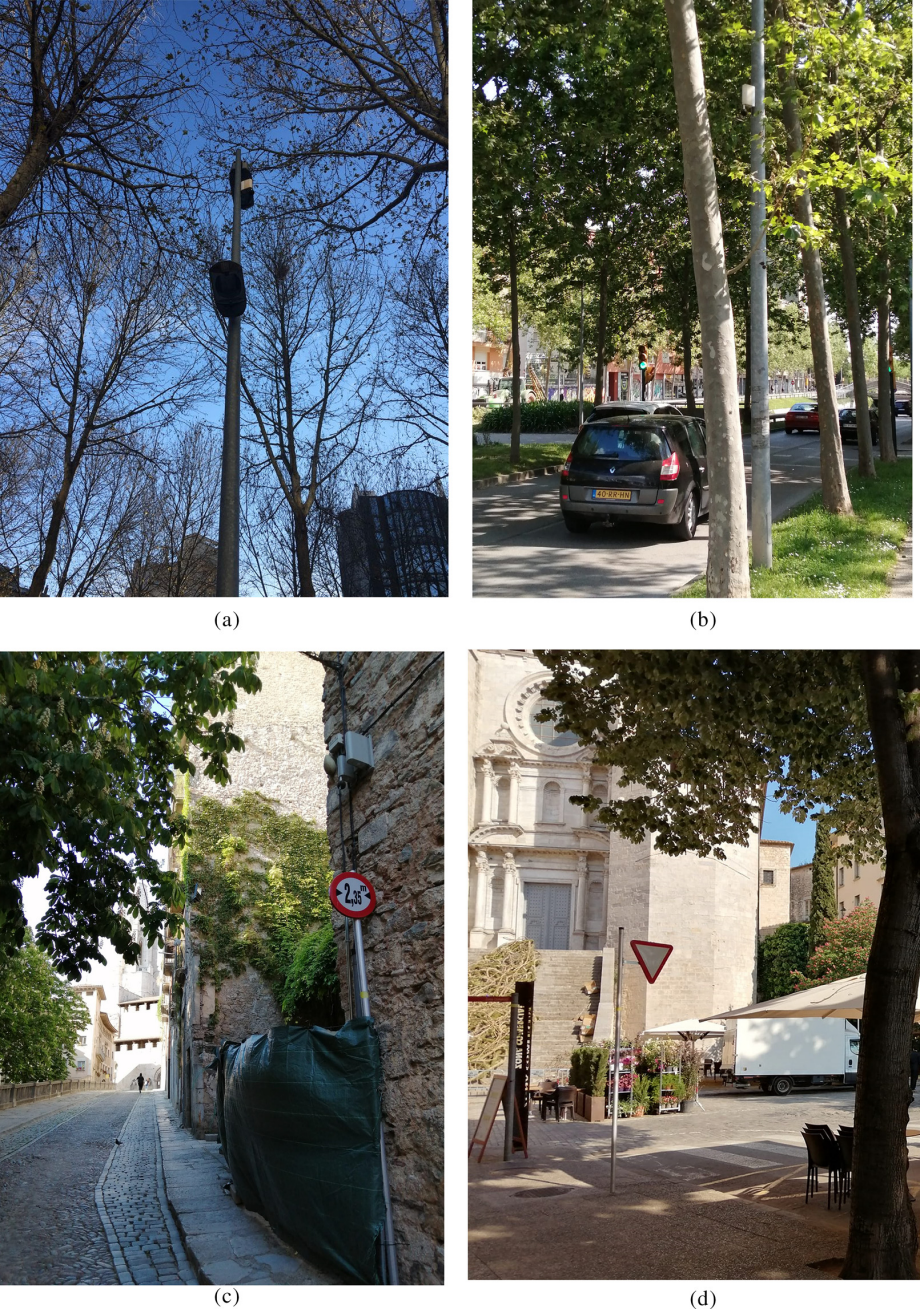
(Color online) Pictures of the four selected sensors in Girona: (a) Ramon Folch #2, (b) Güell #4, (c) Pujada St. Feliu #6, and (d) Plaça St. Feliu #7.

Point #2 (Ramon Folch) corresponds to a city center location, which combines both traffic and leisure noise, and also railway traffic is measured at that point. Point #4 (Güell) is located in a city highway, which makes the place really noisy in terms of traffic. Point #6 (Pj. St Feliu) is located in a quiet touristic zone, in the old quarter. Finally, Point #7 (Pç. St Feliu) is also located in the old quarter, but in a touristic zone with several restaurants and some traffic during the day. For more details, the pictures of the sensors and their surroundings are depicted in Fig. 2. By choosing these four sensors, we detailed four different noise source sensors and guaranteed the completeness of the data, both in 2020 but also in the former two or three years, in order to establish reliable comparisons in terms of *L_Aeq_*.

### A. Daily evolution of $L_{\mathrm{Aeq},10_{min}}$

**FIG. 3.**
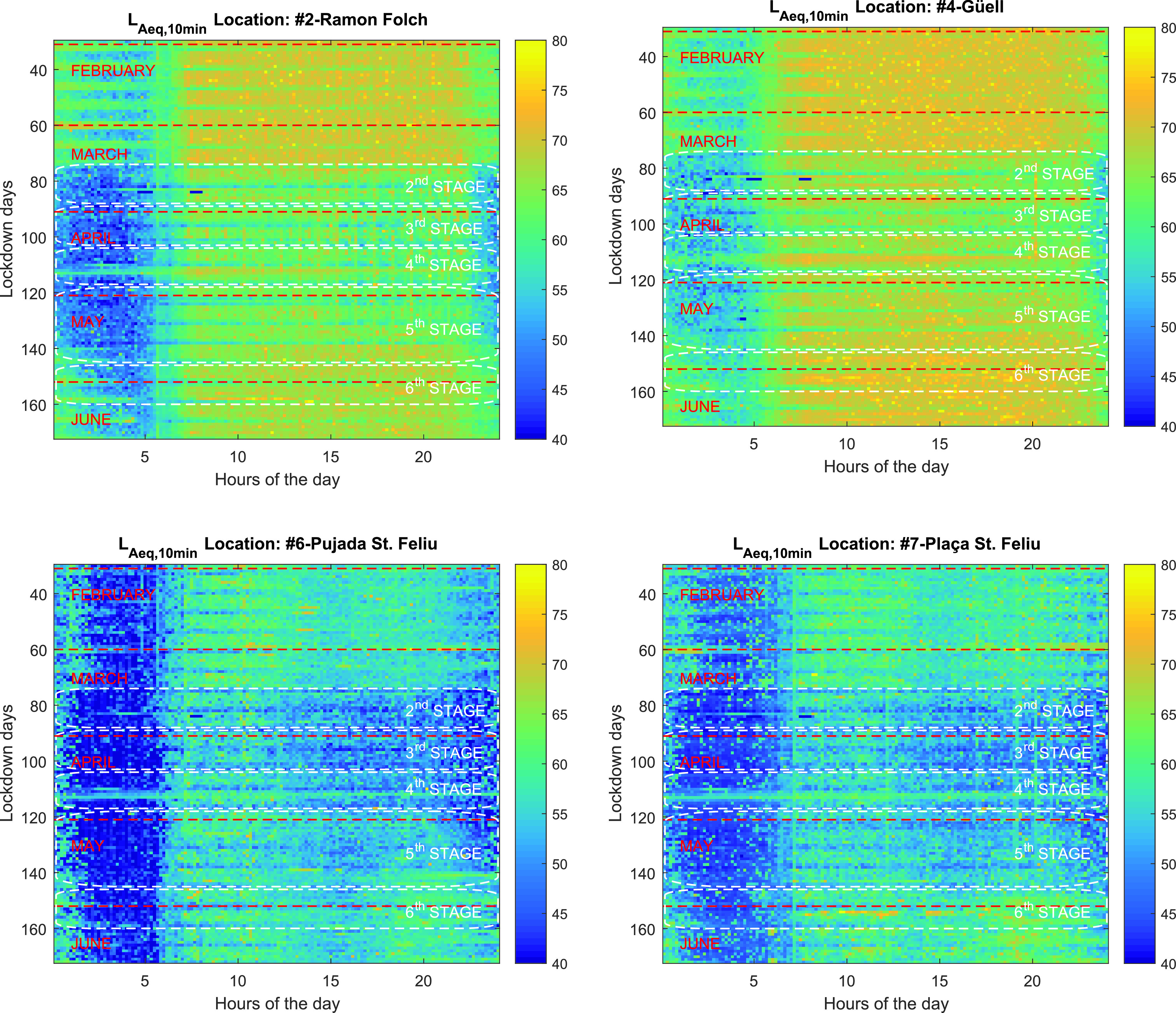
(Color online) $L_{\mathrm{Aeq},10min}$ values for Points #2, #4, #6, and #7. The horizontal axis corresponds to the hour of the day, the vertical axis to the day in 2020 starting from the end of January on the top. The last six different stages of the lockdown are indicated, as well as the month of each row.

In Fig. 3 we plot the time-evolution of the $L_{\mathrm{Aeq},10min}$ values for the four analyzed sensors. The OX axis corresponds to the hours of the day, and the OY axis corresponds to the lockdown days, from top to bottom, starting on January 30th, still with all the February and mid March of pre-lockdown measurements. The plot reaches the end of June, spanning beyond the 6th stage of the lockdown.

Point #2 shows a clear decrease in the $L_{\mathrm{Aeq},10min}$ values, especially during the weekend. Values shown during January, February, and the beginning of March show high values especially during the day, starting mainly at 7–8 a.m. There is a clear difference with the weekend days, periodically drawn as less noisy. Nights in this sensor present complementary values before the lockdown. The noisier ones are for Thursday, Friday, Saturday, and Sunday from each week, due to the fact that it belongs to leisure noise because Ramon Folch is also the center in terms of night activity. During the lockdown, the nights become extraordinary quiet there. An interesting outcome of this sensor is that $L_{\mathrm{Aeq},10min}$ increases, several times per day, with a regular period. Checked with the railway company, it corresponds to the moment that several trains are circulating really close to the sensor (around 7 or 8 a.m.).

Point #4 also shows a decrease in $L_{\mathrm{Aeq},10min}$ when comparing the pre-lockdown period with the lockdown period, but not as deep as the previous sensor. The reason is its location, in a crossroads with high traffic, mainly from the people crossing the city, or entering or going out the city. This traffic comes predominantly from people going to work or going to large supermarkets outskirts of the city, and is probably one of the places in Girona where the traffic was mainly maintained during the lockdown. There is also a decrease during the night, being mostly noticeable during the weekends rather than the week. An interesting conclusion in this sensor is that there is a clear effect of the 8 p.m. tribute to the medical community by clapping at the windows, mainly visible from mid-March until the end of April.

Points #6 and #7 show lower $L_{\mathrm{Aeq},10min}$ values both before the lockdown and during the lockdown. In this sense, the night values of Point #6 present very low values all the nights, and even lower during the lockdown, and moderate day values, and slightly lower during the lockdown, especially from lunchtime on. We have to take into account that it is a pedestrianized zone. Point #7 also shows low night values, but the difference between before the lockdown and during the lockdown is higher. Pç. Sant Feliu presents slightly higher values of $L_{\mathrm{Aeq},10min}$ during the day, probably due to the fact that this is the beginning of the pedestrianized zone in the old quarter, and still keeps some road traffic noise.

Finally, and detailing a global vision, a predominant trend in all four sensors can be seen. The noise decrease between lockdown and pre-lockdown nights is more significant than the reduction of noise contamination between pre-lockdown and lockdown, day and evening. In general, the hours that present a major noise reduction are from 11 p.m. to 7 a.m. if two pandemic and pre-pandemic periods are compared. There is also a slight noise decrease difference between the 2nd and 3rd stage in comparison with the 4th, 5th, and 6th stage.

### B. *L_day_*, *L_eve_*, and *L_night_* values analysis

**FIG. 4.**
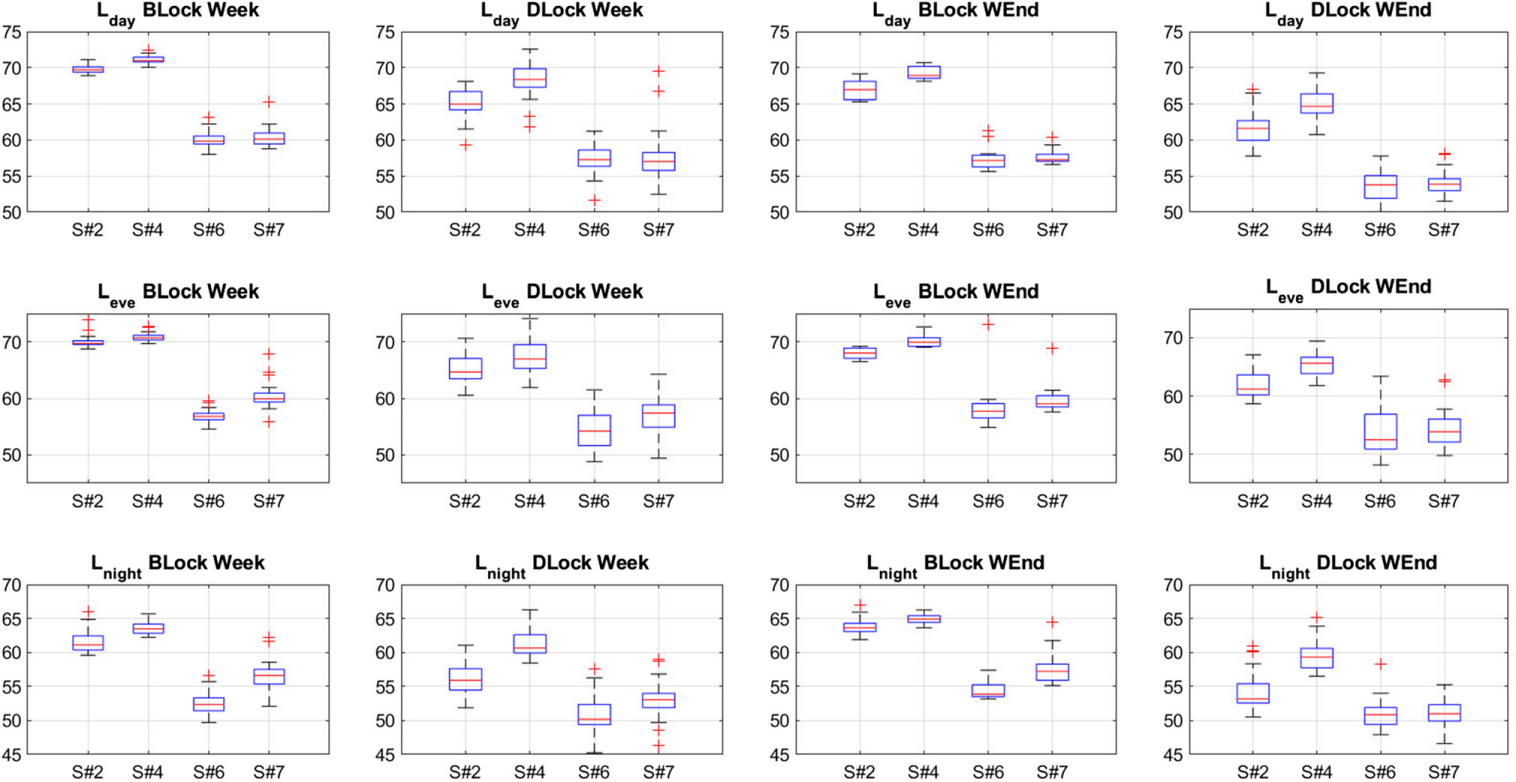
(Color online) *L_day_*, *L_eve_*, and *L_night_* for the four sensors before and during the lockdown and on the week and on the weekend. BLock stands for *before the lockdown* and DLock stands for *during the lockdown*, as well as week stands for *during the week* and WEnd stands for *during the weekend*.

Figure 4 presents the results of the *L_day_*, *L_eve_*, and *L_night_* analysis for the four significant sensors in Girona, split into weekdays and weekend days. *L_day_* has been computed from $L_{\mathrm{Aeq},1min}$, considering noise data between 7 a.m. and 9 p.m.. Moreover, *L_eve_* and *L_night_* have been calculated through $L_{\mathrm{Aeq},1min}$ measures, from 9 p.m. to 11 p.m. and from 11 p.m. to 7 a.m., respectively. The *before the lockdown* analysis (BLock in Fig. 4) starts on January 30th and finishes on March 12th. The *during the lockdown* analysis (DLock in Fig. 4) starts on March 13th and finishes on June 7th.

One of the first conclusions to reach is that Points #2 and #4 are more noisy than Points #6 and #7. *L_day_* values decrease in all the sensors on the weekdays, but especially on the weekend days, where median values of less than 55 dBA can be found in Points #6 and #7. *L_eve_* values also present a decrease during the lockdown, despite, we should note, that Point #4 values keep high presumably due to the tribute to the medical professionals at 8 p.m. Finally, *L_night_* is the most different picture overall for Points #2 and #4, where the leisure noise and road traffic noise are substantially decreased due to the lockdown. Especially, Point #2 has the most significant *L_night_* noise reduction on the weekend, more than 10 dB, which is quite significant. It is also noticeable in Points #6 and #7, but assuming that the noise comes mainly from tourism and restaurants, it did not present high values even before the lockdown.

### C. Hour boxplot comparison

**FIG. 5.**
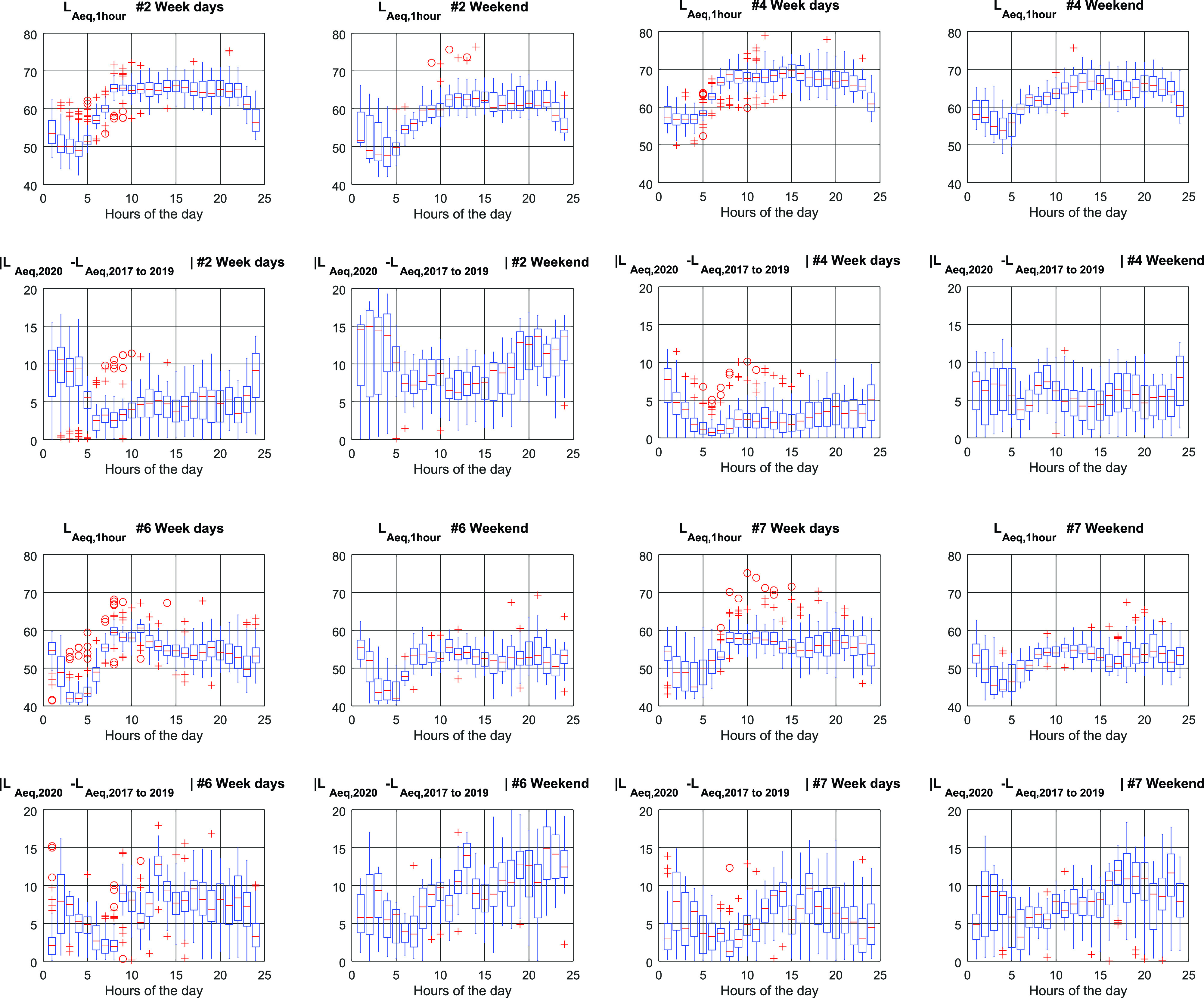
(Color online) Boxplot values for $L_{\mathrm{Aeq},2020}$ and mean $L_{\mathrm{Aeq},2017-2019}$ (the mean value for years 2017, 2018 and 2019) for Points #2, #4, #6, and #7.

In this section, we compare the $L_{eq,1h\mathrm{our}}$ during the 2020 lockdown (i.e., from March 13th to June 7th) with the same period of time throughout the three previous years (i.e., 2017, 2018, and 2019). The first and third rows of Fig. 5 show the $L_{eq,1h\mathrm{our}}$ regarding Points #2 and #4 and Points #6 and #7, respectively, whereas the second and fourth rows show the difference between the $L_{eq,1h\mathrm{our}}$ during the 2020 lockdown and an average of $L_{eq,1h\mathrm{our}}$ throughout 2017, 2018, and 2019 during the same period of the year. In all cases, Fig. 5 plots the results sorted by hours and discriminating between weekdays and weekend days.

Figure 5 shows a behavior for each sensor that can be correlated with the environment where it is placed. Point #2, which is placed in a night leisure area, shows a large difference between the 2020 lockdown period and previous years (i.e., difference in median values higher than 12 dB) at evening and night. Results match with those obtained in Fig. 4 previously. This conduct happens during weekends and with not so high difference median values (i.e., between 7  and 11 dB) also during weekdays. Point #4, which is located in a sort of urban highway where heavy traffic noise almost squelches all remaining noise, shows the lowest difference (i.e., difference in median values always lower than 8 dB) between the 2020 lockdown period and previous years. Point #6, which is placed in a quiet zone in the old quarter of the city, shows the highest difference (median values between 10 and 15 dB) with previous years at weekend evenings. Point #7, which is placed in a location with several restaurants, shows the highest difference with previous years (difference median values between 8 and 12 dB) at afternoon and dinner time mainly at weekends (in Catalonia, dinner time might span from 8 p.m. to 12 a.m.). Furthermore, the trend of noise behaviour during the day can be recognized in each sensor considering noise levels $L_{eq,1h\mathrm{our}}$. Even so, in Points #6 and #7, the difference levels have a more random component, suggesting that there is not a predominant source of environmental noise but several and that they are also variable through hours and days.

## IV. CONCLUSIONS

In conclusion, there is a clear decrease in the noise in the street coming from any of the urban noise sources. The sensors presenting larger differences between the before and during the lockdown period are those that contain night leisure noise, and they also present larger differences with previous years.

Point #2 placed in a night activities area presents noise reduction differences from 5 dB (*L_day_ week*) to 11 dB (*L_night_* weekend), between before and during lockdown noise levels. Similarly, data from Barcelona present an average decrease between 9 and 12 dB for the same typology of sound sources (Ajuntament de Barcelona, 2020). In addition, Madrid data show a lower decrease, between 3.9 (*L_eve_* weekend) and 6.3 dB (*L_night_* weekend) in active areas (Asensio *et al.*, 2020b). In the London study (Aletta *et al.*, 2020b), sound level reduction was 6.6 dB on average between the same periods of 2019 and 2020 in active areas. Active areas are described as those where human activities are the main contributor, combined with traffic noise. Also, the active areas are comparable to where Points #6 and #7 are located. Points #6 and #7 present noise reduction differences from 3  (*L_day_* week) up to 10 dB (*L_night_* weekend).

In comparison, sensors with a heavy charge of traffic present lower differences in all the conducted measurements. For instance, Point #4 presents noise reduction differences between 2  (*L_night_* week) and 8 dB (*L_night_* weekend). Additionally, in Barcelona, noise reduction fluctuates from 2 to 6 dB in heavy traffic highways (Ajuntament de Barcelona, 2020). Traffic noise measurements from Madrid show a similar decrease, between 3.9  (*L_eve_* week) and 7.4 dB (*L_night_* weekend) (Asensio *et al.*, 2020b). Moreover, in Milan and Rome, data show average differences between weekly noise levels in 2019 and 2020 of 7.3 dB (A) *L_den_* and 5.2 dB (A) *L_den_*, respectively (Alsina-Pagès *et al.*, 2020a). Noise level reduction in traffic dominated areas at cities in northernmost areas are comparable. In Paris and London, sound level reduction has been 4.5  and 5.9 dB (A) *L_den_* on average, respectively (Aletta *et al.*, 2020b; Bruitparif, 2020). Other studies have been done in Granada, Spain (Vida Manzano *et al.*, 2021) and Stockholm (Sweden) (Rumpler, Venkataraman, and Goransson, 2021). If we look at absolute noise reduction in Granada between 2019 and 2020, the study shows an impressive difference up to 30 dB (A) in some locations. Data from Stockholm presents a decrease in more than 4 dB (A) on the daily average in April 2020.

In conclusion, the sensors with the highest levels of $L_{eq,1h\mathrm{our}}$, mainly due to traffic noise, are those that experience the lowest differences with previous years. The leisure values decrease especially during the evening and the night, and mainly in the leisure areas. Reduction of night activities, like closing restaurants and bars, has a direct impact on noise contamination reduction mainly in center town areas, as is shown in Point #2.

## Footnotes

^1^ For more information, see http://visoracustic.girona.cat/VisorAcustic/.

^2^ For more information, see https://www.urbiotica.com/.

^3^ For more information, https://www.keacoustics.com/en/home-en/.
